# Differential Influence of COVID-19 pandemic on Life-Space Mobility of older adults

**DOI:** 10.1093/geroni/igab046.3173

**Published:** 2021-12-17

**Authors:** Anna Zisberg, Ksenya Shulyaev, Nurit Gur-Yaish, Efrat Shadmi

**Affiliations:** 1 University of Haifa, Haifa, HaZafon, Israel; 2 Faculty of Social Welfare and Health Science, University of Haifa, Haifa, Hefa, Israel; 3 Oranim Academic College of Education, Haifa, HaZafon, Israel; 4 University of Haifa, Mount Carmel, Israel, Haifa, Hefa, Israel

## Abstract

Life-space mobility (LSM) is critical to aging successfully since it is essential to maintain independence, affecting the health and quality of life of older adults. During the COVID-19 pandemic older adults, who are at high-risk of serious illness and complications, are restricted by stay-at-home orders, limiting their outdoor activities. This study evaluated differences in LSM before and during the pandemic and factors related to limited pre-hospitalization mobility. We used a natural experiment design comparing LSM one month prior to hospitalization and its related factors on two subsamples of hospitalized older adults: recruited before and after February 2020 (pandemic outbreak). No significant differences were observed in LSM between pre-pandemic (N=141, M(SD)=54.9(33,5)) and during-pandemic (N=186, M(SD)=55.3(32.9)) samples, even after adjustment for cognitive, functional, and demographic characteristics (F=2.281, p=0.13). Of those who participated during the pandemic, a total of 94 (50.5%) declared that their mobility was strongly affected by the pandemic and had significantly lower LSM (F=4.626, p<0.01) comparing both to those who declared not being affected (N=92) and to the pre-pandemic group, controlling for potential cofounders. In the "during-pandemic" group older adults with higher basic physical functioning, higher economic status, and those with lower levels of education were more likely to indicate that their pre-hospital mobility was affected by the pandemic. These results show that the pandemic period has a differential effect on life-space mobility of older adults. Functional, socio-economic, and educational factors need to be considered in planning how to maintain older adults’ mobility during the ongoing pandemic.

